# GDF15 participates in epithelial cell senescence in radiation-induced lung injury through the ERK1/2-p16 signaling pathway

**DOI:** 10.1371/journal.pone.0350042

**Published:** 2026-06-09

**Authors:** Jing Liu, Dongyang Lv, Hengjiao Wang, Defu Yang, Ying Xu, Ying Yan

**Affiliations:** 1 Department of Radiation Oncology, General Hospital of Northern Theater Command, Shenyang, China; 2 Graduate School of Dalian Medical University, Dalian, China; Central South University, CHINA

## Abstract

**Objective:**

Radiation-induced lung injury (RILI) is a common complication of thoracic radiotherapy that can compromise treatment outcomes and reduce the quality of life of cancer patients. Cellular senescence is increasingly recognized as an important biological process in the development of RILI. This study aimed to identify senescence-associated molecules involved in RILI and to investigate their potential mechanisms of action.

**Methods:**

Bioinformatics analysis was performed by integrating differential gene expression profiles from a RILI-related GEO dataset with a senescence-related gene set, which identified growth differentiation factor 15 (GDF15) as a candidate molecule of interest. A rat model of RILI was established, and inflammatory injury and fibrosis were evaluated by hematoxylin and eosin (HE) staining and Masson’s trichrome staining. DNA damage was assessed by γH2AX immunofluorescence. Senescence-associated changes were evaluated by senescence-associated β-galactosidase (SA-β-gal) staining and detection of p53, p21, p16, and GDF15 expression by Western blot. In addition, in vivo and in vitro experiments were performed to further explore the potential mechanism associated with GDF15 in radiation-induced epithelial senescence.

**Results:**

Bioinformatics analysis identified GDF15 as a prominently upregulated senescence-related gene in RILI. In irradiated rat lungs, γH2AX expression was significantly increased, accompanied by inflammatory infiltration, fibrotic changes, and upregulation of senescence-associated markers. SA-β-gal staining further supported the presence of radiation-induced senescence in vivo. Similar findings were observed in irradiated BEAS-2B cells. Mechanistic experiments showed that GDF15 knockdown attenuated radiation-induced senescence and downregulated the expression of p-ERK1/2 and downstream p16, while ERK1/2 inhibition reduced senescence-associated β-gal staining and p16 expression. These findings suggest that radiation-induced GDF15 may contribute to epithelial cell senescence during RILI, potentially through the ERK1/2-p16 signaling pathway.

**Conclusion:**

Our findings suggest that GDF15 is upregulated in response to ionizing radiation and may participate in epithelial cell senescence during the development of RILI. This process appears to be associated with the ERK1/2-p16 signaling pathway. These results provide additional insight into the molecular mechanisms underlying RILI and suggest that GDF15 may represent a potential target for future therapeutic intervention.

## Introduction

Radiotherapy is a primary treatment for malignant tumors, particularly lung cancer, playing a vital role in tumor progression control and palliative care [1,2]. Although advances in radiotherapy techniques have reduced the incidence of radiation-induced lung injury (RILI), its adverse effects remain a significant concern. Studies have shown that the incidence of grade ≥3 radiation pneumonitis after 3D-CRT and IMRT in patients with locally advanced non-small cell lung cancer treated with radiotherapy was 7.9% and 3.5%, respectively [3]. However, acute and chronic radiation-induced damage to sensitive lung tissue remains difficult to completely avoid [4]. RILI not only affects the quality of life of patients but can also be life-threatening in severe cases. Previous studies have identified complex cell-to-cell interactions, signaling pathways and dynamic cytokine cascades throughout the RILI process [5]. The main mechanisms of radiation-induced tissue damage are DNA damage and the production of reactive oxygen species (ROS) [6]. Lung irradiation generates a large number of reactive oxygen species and reactive nitrogen species, which lead to oxidative damage to DNA, lipids and proteins. This damage or apoptosis induces inflammatory responses and the chemotaxis of monocytes, lymphocytes, and granulocytes. These cells accumulate at the site of damage, releasing large quantities of inflammatory cytokines and chemokines. When this injury becomes chronic, it eventually leads to pulmonary fibrosis. Overall, numerous cells and molecules are involved in the complex process of radiation-induced lung injury [7].

Senescence is characterized by permanent growth arrest, typically induced by DNA damage or oxidative stress. In the context of RILI, radiation-induced senescence is a key pathogenic factor [8,9]. Ionizing radiation induces a state of permanent growth arrest in alveolar epithelial cell type II (AEC II) through the DNA damage response (DDR) pathway or oxidative stress [10]. Senescent cells secrete a complex mixture of pro-inflammatory cytokines, angiogenic factors, and pro-mitotic molecules, collectively referred to as the senescence-associated secretory phenotype (SASP). This secretion subsequently triggers senescence in previously undamaged alveolar epithelial cells [11]. AEC II, an alveolar stem cell, plays a central role in injury repair and maintains alveolar homeostasis by proliferating and differentiating to regenerate both AEC I and AEC II cells [12]. However, radiation- which in turn will trigger impaired alveolar epithelial repair [13,14], initiating stress injury and fibrosis [15]. The severity of AEC II depletion correlates with the degree of senescence [10], and a significant deficiency of AEC II further recruits macrophages and triggers a cascade of pro-inflammatory factors, ultimately driving the irreversible process of pulmonary fibrosis [14,16]. Growth differentiation factor 15 (GDF15), originally known as macrophage inhibitory factor 1 in 1997, is a stress response cytokine belonging to the transforming growth factor β (TGF-β) superfamily. Also known as nonsteroidal anti-inflammatory drug-induced gene (NAG-1), placental trans forming growth factor – β, prostate-specific factor, and central bone morphogenetic protein [17–19]. As a key protein associated with senescence, GDF15 plays a role in the cellular response to the activation of senescence-related pathways [20].

It is clear that cellular senescence plays a pivotal role in the pathogenesis of RILI. Targeting cellular senescence may therefore present a promising therapeutic strategy for treating this condition. In this study, we integrated the differential expression profiles of senescence-related genes and RILI through bioinformatics, screened for core regulatory molecules-GDF15, and systematically examined their roles in radiation-induced lung epithelial cell senescence, inflammation, and fibrosis. This was done by combining the rat model of RILI with cellular experiments, with the goal of identifying new therapeutic targets and providing a theoretical basis for clinical intervention in RILI.

## Materials and methods

### Data acquisition and identification of differential genes

The gene expression data analyzed in this study were obtained from Gene Expression Omnibus (GEO) database (https://www.ncbi.nlm.nih.gov/geo/). The datasets GSE25295, GSE14431, GSE41789, GSE85359 were downloaded from GEO and used for subsequent bioinformatics analyses. Both control and radiation exposure data were included in these datasets. Data processing was performed using R software, and principal component analysis (PCA) was conducted to evaluate the reproducibility of the GSE datasets. The ‘SVA’ R package was used to adjust for batch effects between datasets, ensuring more reliable comparisons. Statistical significance was determined with an adjusted p-value < 0.05 and a log fold change |logFC| > 0.585. Volcano plots and heatmaps of differentially expressed genes were generated using R software. The dataset of senescence-associated genes was retrieved from the CellAge database, and the senescence-related differential genes were identified by intersecting them with the screened differential genes.

### Bioinformatics analyses

To explore the possible biological functions and mechanisms of genes, Gene Ontology (GO), Kyoto Encyclopedia of Genes and Genomes (KEGG) analyses were performed. Protein-protein interaction (PPI) networks were constructed using the STRING database (http://www.string-db.org) and visualized using Cytoscape software. Additionally, the Cytoscape plugin cytoHubba was used to identify key hub genes within the PPI network. The R package was used to assess the correlations between differentially expressed senescence-related genes.

### Animals and experimental grouping

Male Sprague-Dawley (SD) rats were acquired from Liaoning Changsheng Biotechnology Co., Ltd. All experimental procedures were approved by the General Hospital of Northern Theater Command Animal Care Committee and conducted in strict compliance with Institutional Animal Care and Use protocols. All rats were housed in groups at room temperature (24 ± 2°C) under a 12-hour light/12-hour dark cycle, with free access to water and food. Rats were randomly assigned to a control group and an irradiation group, with six rats in each group at each time point. Radiation doses were determined based on previous studies [21]. In the irradiation group, rats were exposed to 6MV X-rays in the right lung at a dose rate of 300 cGy/min, with a total irradiation dose of 30 Gy. Rats were anaesthetised via intraperitoneal injection of sodium pentobarbital to minimise discomfort during the irradiation process. The rats were euthanized by intraperitoneal injection of 150 mg/kg pentobarbital sodium on the 3rd, 7th, 14th, and 28th days after irradiation, and lung tissue specimens were collected. The death is confirmed by no respiration, pulse, heartbeat and no nerve reflexes for more than 5 min. All necessary efforts were made to minimize the suffering. Subsequent experimental studies will be conducted on tissues collected 28 days after radiation exposure.

### HE staining

Lung tissues were fixed in 4% paraformaldehyde, followed by dehydration, clearing, and embedding in paraffin. Sections (5μm thick) were prepared, dewaxed, and rehydrated. After staining with hematoxylin and eosin, the sections were dehydrated, cleared, and sealed with neutral gum. The pathological changes in lung tissue were examined under a light microscope.

### Masson staining

Rat lung tissues, fixed in 4% paraformaldehyde, were subjected to routine paraffin embedding and Masson’s trichrome staining. After differentiation, clearing, and sealing, collagen fibers were observed under a light microscope.

### Immunofluorescence

Frozen tissue sections were thawed, fixed, permeabilized, and blocked. Sections were incubated with diluted primary antibody at 4°C overnight. The next day, sections were washed with PBS, incubated with the secondary antibody at room temperature for 1 hour, and counterstained with DAPI. The sections were then sealed with an anti-fluorescence quenching agent, and images were captured using a fluorescence microscope.

### Western blotting

Cells and lung tissues were homogenized in RIPA buffer containing protease and phosphatase inhibitors using a tissue grinder. Protein concentration was measured using the BCA protein concentration kit. Protein samples were separated by SDS-PAGE and transferred to nitrocellulose membranes, which were blocked with 5% skim milk. Membranes were incubated overnight with the relevant primary antibody, followed by incubation with a horseradish peroxidase-conjugated secondary antibody. Immunoblotting signals were detected using enhanced chemiluminescence. The primary antibodies used were as follows: γH2AX (CST,USA), γH2AX(CST,USA) p21 (Invitrogen, USA), p16 (Invitrogen, USA), GDF15 (Proteintech, China), IL-6 (Proteintech, China), IL-1β Proteintech, China), IL-18 (Proteintech, China), TNF-α(Santa Cruz, USA), TGF-β(Proteintech, China), MCP1(Proteintech, China), ERK1/2(Proteintech, China), p-ERK1/2(Proteintech, China), MMP2(SantaCruz, USA), MMP9(SantaCruz, USA), β-actin(Servicebio, China), α-Tubulin (ABclonal, China).

### SA-β-gal staining

SA-β-gal staining was performed using the SA-β-gal Staining Kit (Beyotime, China). Cells and frozen tissue sections were washed 3 times with PBS, fixed with fixative for 15 minutes at room temperature, and washed again with PBS. Fresh SA-β-gal staining working solution was prepared according to the manufacturer’s instructions and added to the samples. The samples were then incubated at 37°C in a CO_2_-free atmosphere overnight, and staining was observed under a light microscope.

### Transcriptomic sequencing

RNA was extracted from rat lung tissue and subjected to precise assessment of integrity and total quantity using the Agilent 2100 Bioanalyzer. mRNA was enriched from total RNA via Oligo dT magnetic beads to construct the library. Libraries were quantified using a Qubit instrument and real-time fluorescent quantitative PCR, with fragment size distribution analyzed via bioanalyzer. Following successful library quality assessment, Illumina sequencing was performed to obtain sequence data.

### Cell culture and irradiation

The human bronchial epithelial cell line BEAS-2B cells were purchased from the Cell Bank of the Chinese Academy of Sciences (Beijing, China), and were cultured in DMEM medium supplemented with 10% fetal bovine serum, 1% penicillin and streptomycin. The culture was maintained at 37°C in a 5% CO_2_ atmosphere. The medium was replaced every 2–3 days, and cells were passaged when the confluence reached 80%. Establish a cellular model based on previous research [22]. Irradiate cells when they reach the desired density. The irradiation dose is 10 Gy. Extract proteins or perform other experiments 48 hours after irradiation.

### ELISA analysis

Cell culture supernatant was collected for ELISA analysis. Prepare reagents according to the GDF15 ELISA Kit (UpingBio, China) instructions. Measure absorbance at 450 nm using a microplate spectrophotometer. Plot a standard curve and calculate GDF15 concentrations.

### siRNA transfection

To inhibit GDF15 expression, BEAS-2B cells were seeded in 6-well plates. GDF15 siRNA was transfected into BEAS-2B cells using a transfection reagent (Sangon China) according to the manufacturer’s instructions. Cellular proteins were extracted after culture for subsequent analysis. The siRNA sequences were as follows: 5’ to 3’ Sense: CCUCAGAGUUGCACUCCGA; Antisense: UCGGAGUGCAACUCUGAGG.

### Statistical analysis

Statistical analysis was conducted using GraphPad Prism 8.0 software. All data are presented as mean ± standard deviation (SD). The Student’s t-test was used to compare two groups, while two-way analysis of variance (ANOVA) was applied for comparisons among multiple groups. A p-value of < 0.05 was considered statistically significant.

## Results

### Identification of senescence-related differentially expressed genes

**Fig 1:**
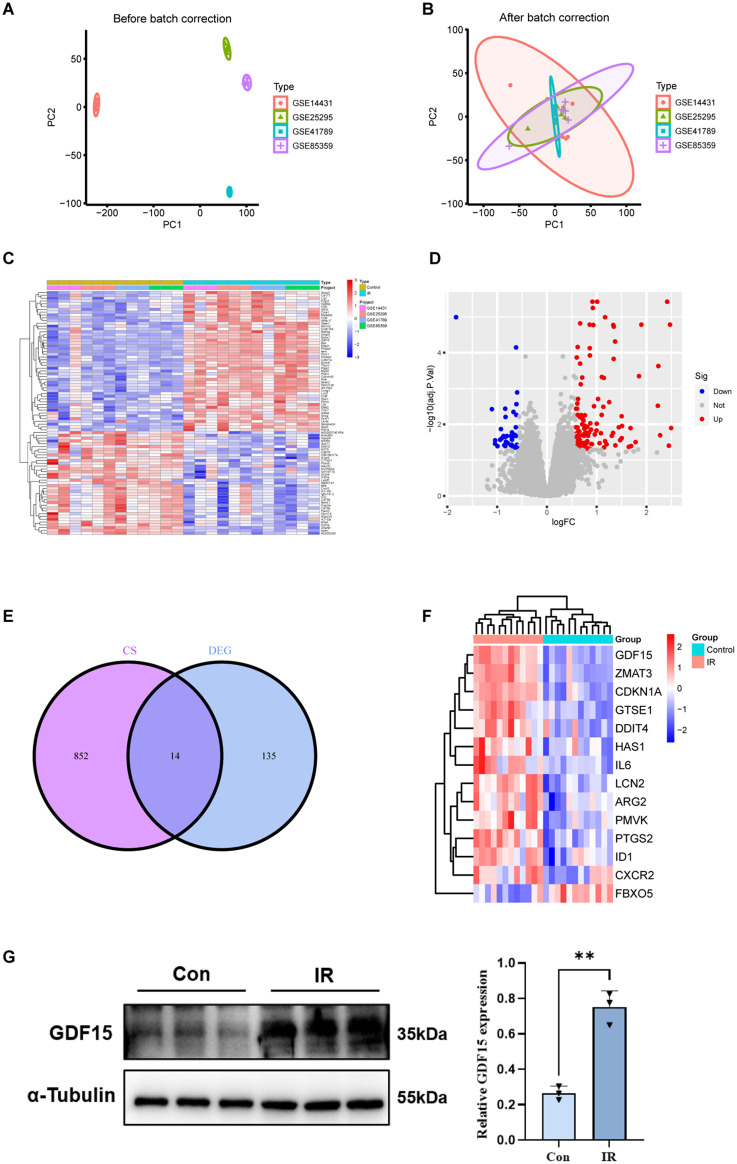
Identification of senescence-related differentially expressed genes. **(A)** PCA before batch-effect correction. **(B)** PCA after batch-effect correction. **(C)** Heatmap of differential expressed genes (DEGs). **(D)**Volcano plot of DEGs. **(E)** Venn diagram of DEGs and cellular senescence-related genes. **(F)**Heatmap of senescence-related DEGs. **(G)** Representative western blot images and quantitative analysis of GDF15 expression (n = 3). *P < 0.05, **P < 0.01, ***P < 0.001.

After batch-effect correction, principal component analysis (PCA) showed improved separation between groups, indicating that the datasets were suitable for subsequent integrated analysis (Fig 1A-B). Differential expression analysis identified two sets of differentially expressed genes (DEGs), which are shown in the heatmap and volcano plot (Fig 1C-D). By intersecting these DEGs with a senescence-related gene set, 14 senescence-related DEGs were identified (Fig 1E). Among them, GDF15 showed the most prominent differential expression (Fig 1F). To validate this bioinformatics finding, GDF15 expression in rat lung tissue was examined by Western blot, which confirmed significant upregulation of GDF15 in the irradiated group (Fig 1G).

### Functional enrichment analysis

**Fig 2:**
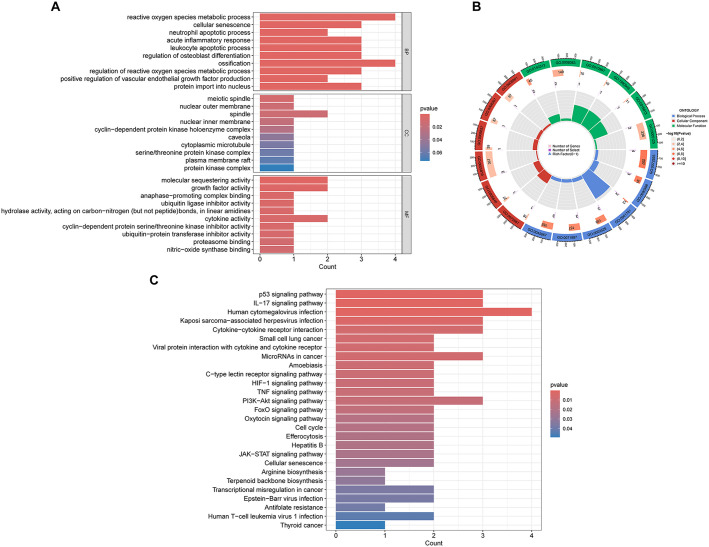
Enrichment Analysis. **(A)** Enrichment analysis of senescence-related DEGs in BP, CC and MF. **(B)** Circular plot depicting GO enrichment of senescence-related DEGs. **(C)** KEGG enrichment analysis of senescence-related DEGs.

GO and KEGG enrichment analyses were performed to explore the potential biological significance of the identified senescence-related DEGs. GO analysis revealed significant enrichment in biological processes associated with reactive oxygen species metabolism and cellular senescence, indicating that oxidative stress and senescence may be major biological features of RILI. In terms of cellular components, the DEGs were mainly associated with spindle-related structures, while molecular function analysis indicated enrichment in molecular sequestering activity and growth factor activity. KEGG pathway analysis further demonstrated significant enrichment in the cellular senescence pathway and the p53 signaling pathway (Fig 2A–C). Collectively, these findings highlighted the involvement of senescence-related signaling in radiation-induced lung injury and provided a rationale for subsequent analysis of GDF15.

### Protein-protein interaction network (PPI) and Co-expression analysis

**Fig 3:**
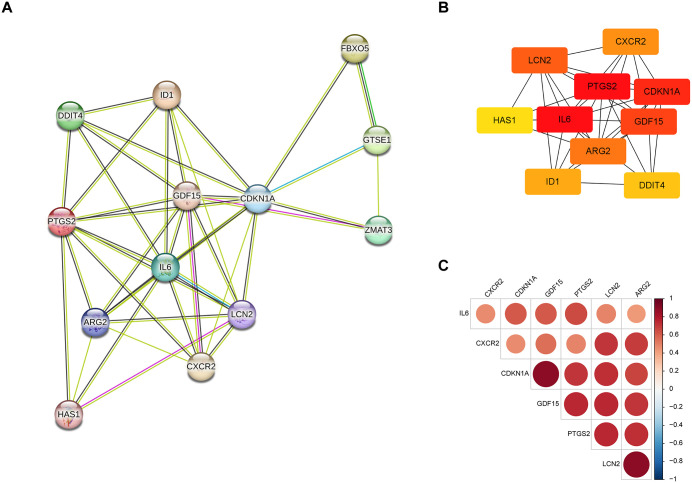
PPI analysis the of senescence-related DEGs. **(A)** PPI networks of senescence-related DEGs. **(B)** Subnetwork of the top 10 hub genes. **(C)** Correlation heatmap of senescence-related DEGs.

A protein-protein interaction (PPI) network was constructed using the STRING database and visualized with Cytoscape to identify key regulatory molecules among the senescence-related DEGs. Ten hub genes were identified using the cytoHubba plugin, including IL-6, CDKN1A, and GDF15 (Fig 3A-B). These hub genes are closely related to inflammatory and senescence-associated pathways, suggesting that they may function as central regulators in the response to radiation exposure. In addition, Spearman correlation analysis demonstrated significant correlations among several of the identified genes (Fig 3C), revealing an interconnected senescence-associated regulatory network in RILI.

### Radiation-induced alveolar inflammation and fibrosis in rats

**Fig 4:**
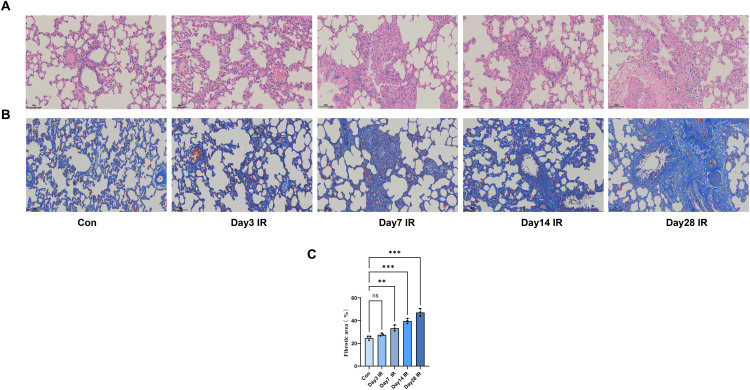
Establishment of the radiation-induced lung injury model. **(A)** HE staining of lung tissue (magnification×200) (n = 3). **(B)** Masson’s trichrome staining of lung tissue (n = 3). **(C)** Quantitative analysis of the fibrotic area in lung tissue (magnification×200) (n = 3). *P < 0.05, **P < 0.01, ***P < 0.001.

HE staining revealed that the alveolar walls in the lung tissue were thin, with clear alveolar structures, intact capillaries, and no signs of inflammatory cell infiltration. In the model group, lung tissue primarily displayed exudative lesions, pronounced interstitial edema, enlarged alveolar septa, and inflammatory cell infiltration around the bronchioles (Fig 4A). Masson staining further highlighted significant collagen fiber proliferation in the model group ( Fig 4B and 4C), consistent with fibrotic remodeling after radiation exposure.

### Radiation-induced DNA damage and senescence in rat lung tissue

**Fig 5:**
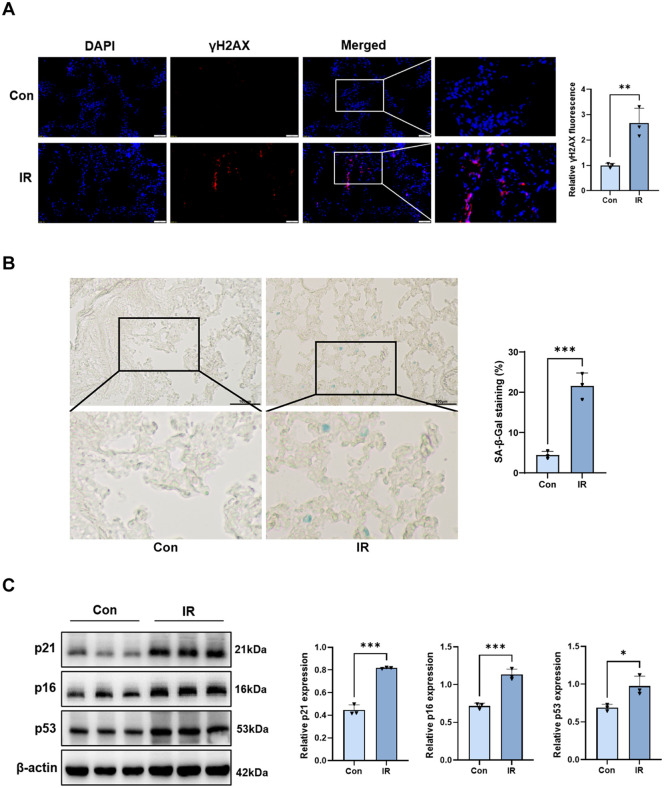
Radiation-induced DNA damage and cell senescence. **(A)** Immunofluorescence staining for γH2AX (red), DAPI (blue) and quantitative analysis of relative γH2AX fluorescence intensity in lung tissue (n = 3). **(B)** Representative SA-β-gal staining images and quantitative analysis. **(C)** Representative western blot images and quantitative analysis of p21, p16 and p53 (n = 3).

Immunofluorescence staining showed a significant increase in γH2AX in irradiated lungs compared with the control group, indicating enhanced DNA damage after radiation exposure (Fig 5A). We next evaluated classical senescence-associated markers. To provide more direct evidence of senescence in vivo, we further performed SA-β-gal staining in lung tissue sections. The number of SA-β-gal-positive cells was markedly increased in irradiated lungs compared with controls (Fig 5B), which further confirmed the presence of radiation-induced cellular senescence in the animal model. Western blot analysis revealed significantly increased expression of p53, p21, and p16 in the irradiated group (Fig 5C), suggesting activation of the senescence program.

### Radiation-induced SASP-related inflammation and fibrosis

**Fig 6:**
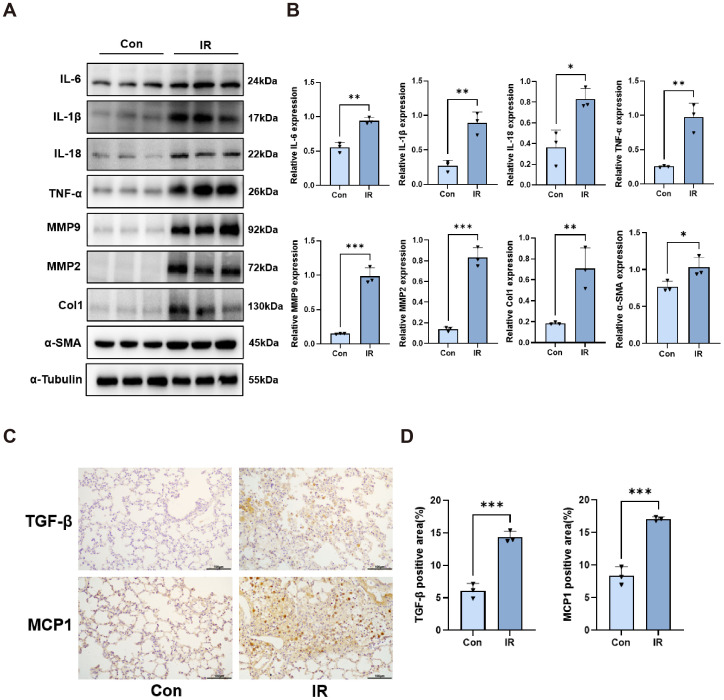
Radiation-induced SASP-related inflammation and fibrosis. **(A-B)** Representative western blot images and quantitative analysis of IL-6, IL-1β, IL-18, TNF-α, MMP9, MMP2, Col1 and α-SMA (n = 3). **(C-D)** Immunohistochemical staining and quantitative analysis of TGF-β and MCP1 in lung tissue (magnification×200) (n = 3). *P < 0.05, **P < 0.01, ***P < 0.001.

SASP-related inflammatory mediators and matrix-remodeling factors were examined to assess the biological consequences of cellular senescence. The irradiated group showed increased expression of IL-6, IL-1β, IL-18, TNF-α, MMP9, MMP2, Col1 and α-SMA (Fig 6A-B), indicating that radiation-induced senescence was accompanied by a pro-inflammatory and matrix-remodeling microenvironment. In addition, immunohistochemistry showed increased expression of TGF-β and MCP1 in lung tissue (Fig 6C-D), consistent with a profibrotic and proinflammatory response.

### Transcriptomic sequencing of rat lung tissue

**Fig 7:**
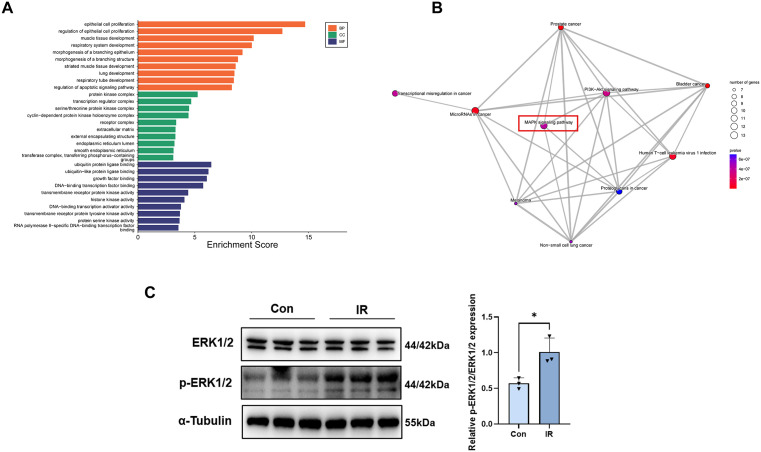
Transcriptomic sequencing of rat lung tissue. **(A)** GO analysis of senescence-related DEGs. **(B)** KEGG analysis of senescence-related DEGs. **(C)** Representative western blot images and quantitative analysis of p-ERK1/2 and ERK1/2 in lung tissue (n = 3). *P < 0.05, **P < 0.01, ***P < 0.001.

To systematically elucidate comprehensive transcriptional alterations in lung tissue following radiation-induced injury, we performed high-throughput transcriptome sequencing on lung tissue from irradiated and control rats. We performed GO and KEGG analyses by intersecting the transcriptomic sequencing results with aging-related genes. GO analysis of the intersecting genes revealed that primary involvement in epithelial cell proliferation, cyclin-dependent protein kinase holoenzyme complex, and DNA-binding transcription activator activity (Fig 7A). KEGG enrichment analysis demonstrated significant enrichment of the MAPK signaling pathway, which is closely associated with cellular senescence (Fig 7B). Because ERK1/2 is a major branch of MAPK signaling and is known to regulate senescence-related pathways, we further examined ERK1/2 activation in lung tissue. Western blot analysis showed increased phosphorylation of ERK1/2 in irradiated lungs (Fig 7C). Together with the transcriptomic findings, this pointed to a potential role of MAPK/ERK1/2 signaling in radiation-induced senescence and provided a basis for subsequent in vitro validation.

### Radiation-induced cellular senescence of BEAS-2B cells

**Fig 8:**
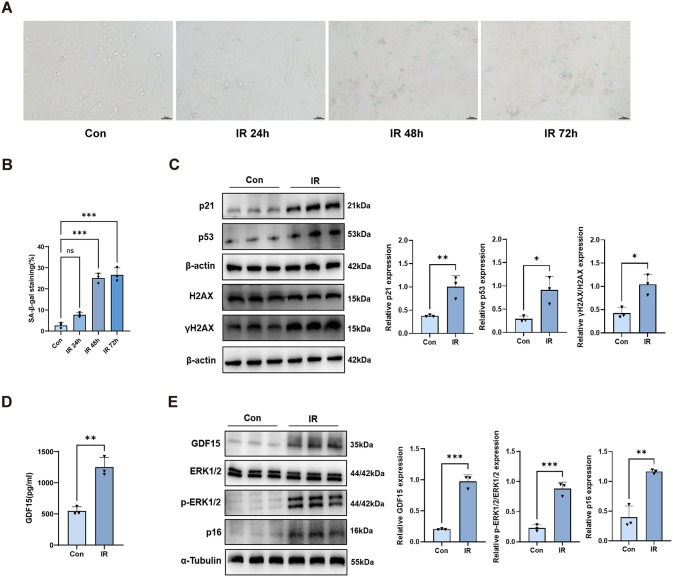
Radiation-induced cellular senescence in BEAS-2B cells. **(A-B)** Representative SA-β-gal staining images and quantitative analysis in BEAS-2B cells (n = 3). **(C)** Representative western blot images and quantitative analysis of p21, p53, H2AX and γH2AX (n = 3). **(D)** ELISA analysis of GDF15 concentration. **(E)** Representative western blot images and quantitative analysis of GDF15 and p-ERK1/2, ERK1/2 and p16 (n = 3). *P < 0.05, **P < 0.01, ***P < 0.001.

Radiation-induced DNA damage and cellular senescence were examined in BEAS-2B cells exposed to 10 Gy, with β-galactosidase staining performed at 24, 48, and 72 h post-irradiation. Results demonstrated a marked increase in senescence staining post-irradiation (Fig 8A-B), accompanied by elevated expression levels of senescence markers including p53 and p21 proteins (Fig 8C). Furthermore, ELISA and Western blot analyses of cell supernatants revealed significantly increased expression of GDF15, phosphorylated ERK1/2 and p16 following radiation exposure (Fig 8D-E), suggesting a possible association between GDF15 and ERK1/2 activation during radiation-induced epithelial senescence.

### GDF15 regulates radiation-induced cellular senescence through the ERK1/2-p16 signaling pathway

**Fig 9:**
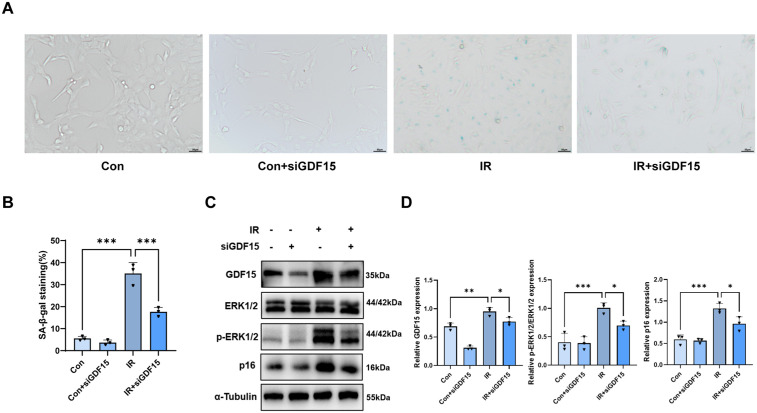
GDF15 regulates radiation-induced cellular senescence through the ERK1/2-p16 signaling pathway. **(A-B)** Representative SA-β-gal staining images and quantitative analysis in BEAS-2B cells (n = 3). **(C-D)** Representative western blot images and quantitative analysis of GDF15, p-ERK1/2, ERK1/2 and p16 (n = 3). *P < 0.05, **P < 0.01, ***P < 0.001.

The role of GDF15 in radiation-induced epithelial senescence was assessed by transfecting BEAS-2B cells with GDF15 siRNA after irradiation. Compared with the radiation-only group, GDF15 knockdown significantly reduced SA-β-gal staining, indicating attenuation of the senescent phenotype (Fig 9A and 9B). In parallel, Western blot analysis showed that GDF15 silencing decreased phosphorylation of ERK1/2 and reduced expression of the downstream senescence marker p16 ( Fig 9C and 9D). These findings indicated that GDF15 may participate in radiation-induced epithelial senescence in association with the ERK1/2-p16 pathway.

### ERK1/2 inhibition attenuates radiation-induced epithelial senescence

**Fig 10:**
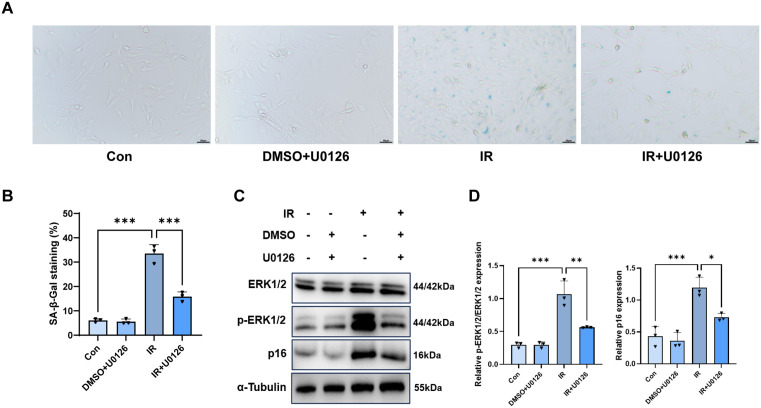
ERK1/2 inhibition attenuates radiation-induced epithelial senescence. **(A-B)** Representative SA-β-gal staining images and quantitative analysis in BEAS-2B cells (n = 3). **(C-D)** Representative western blot images and quantitative analysis of p-ERK1/2, ERK1/2 and p16 (n = 3). *P < 0.05, **P < 0.01, ***P < 0.001.

To further confirm the involvement of ERK1/2 signaling in radiation-induced senescence, BEAS-2B cells were treated with the ERK1/2 inhibitor U0126 after irradiation. ERK1/2 inhibition significantly reduced SA-β-gal positivity and decreased p16 expression (Fig 10A-D). These results support the notion that ERK1/2 signaling contributes to radiation-induced epithelial senescence and further validate the mechanistic link between GDF15 and the ERK1/2-p16 pathway.

## Discussion

RILI is a common complication of radiotherapy, which significantly impairs oncological treatment outcomes and diminishes the quality of life of cancer patients [23]. Extensive evidence suggests that the pathological process of RILI involves multiple biological cascades triggered by ionizing radiation, including DNA damage, oxidative stress, cellular senescence, and inflammatory network imbalances, ultimately leading to acute pneumonitis and progressive fibrosis [24]. RILI is accompanied by direct DNA damage and ROS production [25]. Clinical data show that 43% of patients exhibit imaging features of lung damage after treatment [26]. Previous studies have shown that early apoptosis and persistent cellular senescence occur in alveolar type II cells following radiation exposure [27]. Senescent cells hinder tissue repair and exhibit hallmark changes in the senescence-associated secretory phenotype, including transcriptional, epigenetic, morphological, and metabolic alterations [28]. Inflammatory cell infiltration, cytokine imbalances, and oxidative stress contribute both directly and indirectly to the progression of RILI. Senescent cells display abnormal metabolic activity [29], along with morphological, biological, and genetic changes. Increased β-galactosidase activity, a key senescence marker [30], correlates with G1/S phase cell cycle arrest [31]. In addition, the expression of cell-cycle inhibitors such as p16^INK4a and p21 is elevated, leading to inhibition of cyclin-dependent kinase activity and subsequent cell-cycle arrest [32].

GDF15 is a member of the transforming growth factor-β superfamily [17]. Its expression is upregulated in various pathological conditions, including inflammation, aging, cancer [33], oxidative stress, and hypoxia [34–36]. A single-cell sequencing study identified lung epithelial cells as the primary source of GDF15 in the lungs [37]. In a bleomycin-induced lung fibrosis model, GDF15 expression increased in bronchoalveolar lavage fluid and plasma, correlating with cellular senescence markers in alveolar epithelial cells. Furthermore, GDF15 has been shown to exert pro-fibrotic effects by activating fibroblasts and M2 macrophages [38]. Our study also found that GDF15 is involved in lung tissue remodeling by regulating the cellular senescence program early in radiation-induced injury. In an animal model with telomere dysfunction in type 2 alveolar epithelial cells, GDF15 was the most significantly upregulated protein. Additionally, GDF15 expression is elevated in human idiopathic pulmonary fibrosis (IPF) lungs [37]. It has been suggested that elevated GDF15 levels in blood may precede the development of pulmonary fibrosis and may mediate the link between cellular senescence and interstitial lung abnormalities [39]. Consistent with these observations, our study demonstrated that GDF15 expression was significantly increased in lung tissues of irradiated rats, accompanied by inflammatory infiltration and fibrotic changes. At the same time, we observed increased expression of several senescence-associated markers, including p53, p21, p16, and β-galactosidase. These findings suggest that GDF15 may participate in lung tissue remodeling and cellular senescence during RILI. ERK1/2 is a key regulator of intra- and extracellular signaling, playing an essential role in processes such as cell proliferation, differentiation, cytoskeletal organization, and cellular senescence [40]. Previous studies have reported that GDF15 induces cell proliferation through the PI3K/Akt and ERK signaling pathways [41]. In our study, transcriptome analysis indicated enrichment of MAPK-related signaling pathways after radiation exposure, indicating that the MAPK/ERK1/2 signaling pathway may be involved in the senescence response during RILI. Silencing GDF15 in BEAS-2B cells can significantly alleviate radiation-induced senescence, while reducing the phosphorylation of ERK1/2 and the expression of its downstream effector p16. This indicates that the ERK1/2-p16 axis is a key signaling cascade involved in the pro senescence effect of GDF15 in lung epithelial cells. Notably, our findings align with and extend previous research in endothelial cells, which established that GDF15 induces cellular senescence through a ROS-mediated p16 pathway [42]. In summary, we found that ionizing radiation induces upregulation of GDF15 in lung epithelial cells, and the increase of GDF15 may promote the activation of ERK1/2 signaling, thereby promoting cellular senescence associated with p16.

While our study provides additional insights into the role of GDF15 in RILI, several limitations should be acknowledged. First, the present study primarily focused on epithelial cell senescence, whereas the direct effects of GDF15 on other relevant cell types, such as fibroblasts and macrophages, were not systematically investigated. Given that GDF15 is a secreted cytokine, it may exert paracrine effects within the lung microenvironment and thereby contribute to fibrosis and inflammation through additional cellular pathways. Second, although fibrotic changes were supported by histological and molecular evidence, the extent to which GDF15-induced epithelial senescence directly drives fibrotic progression and overall lung injury severity remains unclear. Further studies will be needed to clarify the contribution of epithelial senescence to fibrotic remodeling and to define its functional impact on the progression of RILI.

## Conclusion

In summary, our findings indicate that GDF15 is involved in radiation-induced lung cell senescence and suggest that its mechanism of action may involve the activation of the ERK1/2-p16 signaling pathway. These results provide additional insights into the molecular basis of RILI and suggest that GDF15 may be a potential target for future therapeutic interventions.

## Supporting information

S1 File The original blots.
(PDF)

S1 Table The senescence-related DEGs in transcriptomic sequencing.
(XLSX)
