# Correction: GDF15 participates in epithelial cell senescence in radiation-induced lung injury through the ERK1/2-p16 signaling pathway

**DOI:** 10.1371/journal.pone.0356407

**Published:** 2026-08-17

**Authors:** Jing Liu, Dongyang Lv, Hengjiao Wang, Defu Yang, Ying Xu, Ying Yan

There are errors in the author affiliations. The correct affiliations are as follows,

Jing Liu^1,2^, Dongyang Lv^1^, Hengjiao Wang^1^, Defu Yang^1^, Ying Xu^1^, Ying Yan^1^

**1** Department of Radiation Oncology, General Hospital of Northern Theater Command, Shenyang, China, **2** Graduate School of Dalian Medical University, Dalian, China.
