# Supplementary material for: GDF15 participates in epithelial cell senescence in radiation-induced lung injury through the ERK1/2-p16 signaling pathway
Source: PLoS One. 2026 Jun 9;21(6):e0350042. doi: 10.1371/journal.pone.0350042 (PMC13249152; doi:10.1371/journal.pone.0350042)
Supplement: S1 File — The original blots. (PDF) [file pone.0350042.s001.pdf]

Lung tissue

p21	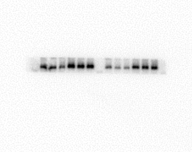	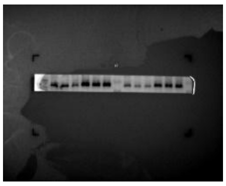	β-actin		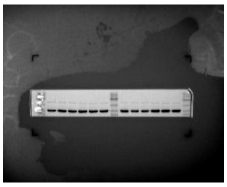
p16	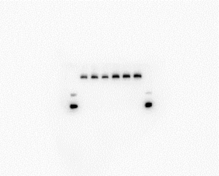	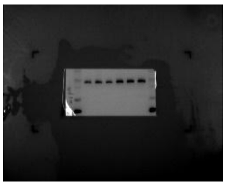	β-actin	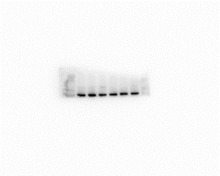	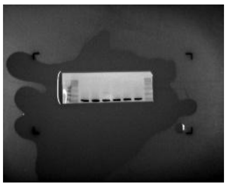
p53	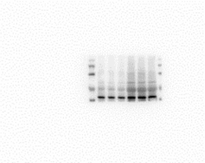	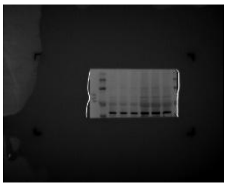	β-actin		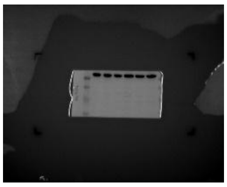
IL-6	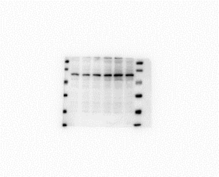	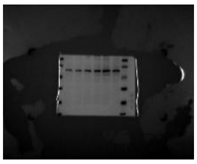	α-Tubulin		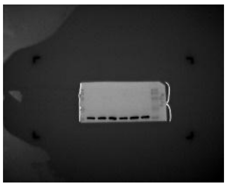
IL-1β	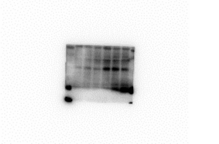	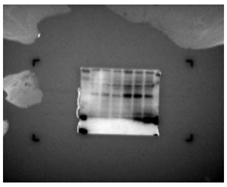	α-Tubulin		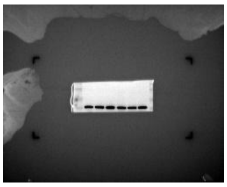
IL-18	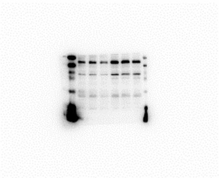	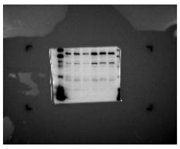	α-Tubulin		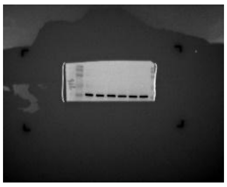
TNF-α	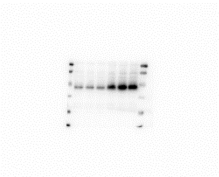	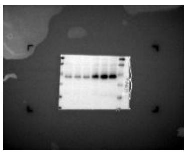	α-Tubulin		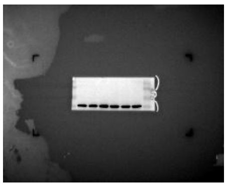
MMP9		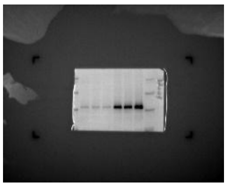	α-Tubulin		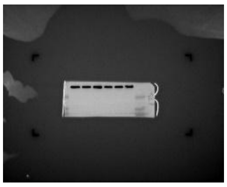

MMP2	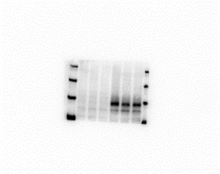	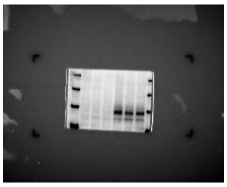	α-Tubulin		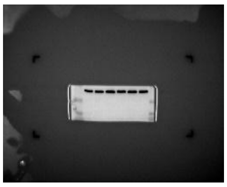
Col1	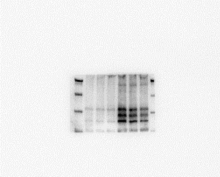	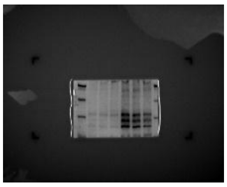	α-Tubulin		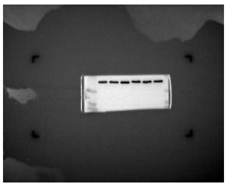
α-SMA		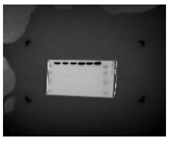	α-Tubulin		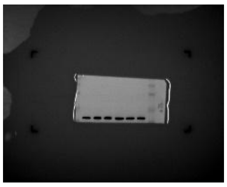
GDF15	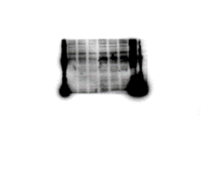	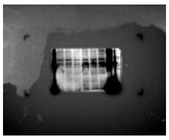	α-Tubulin		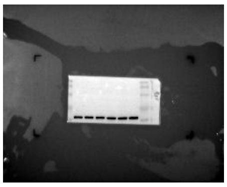
ERK1/2		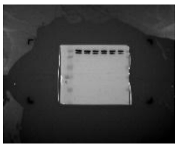	α-Tubulin	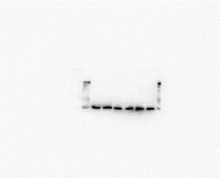	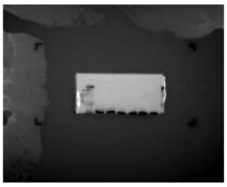
p-ERK1/2	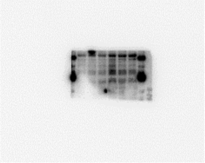	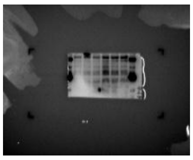	α-Tubulin		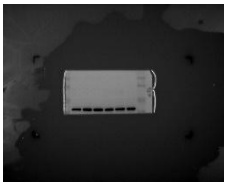

BEAS-2B cell

p21	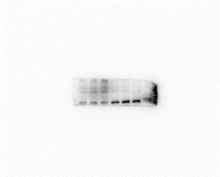	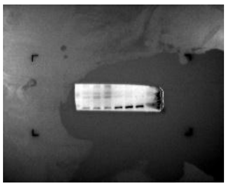	β-actin		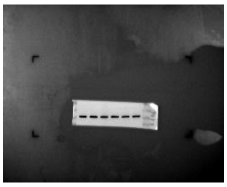
p16	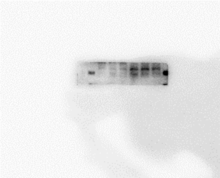	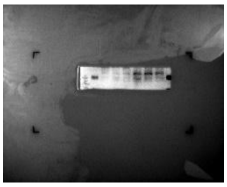	β-actin		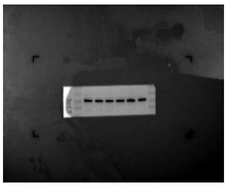
p53	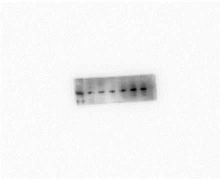	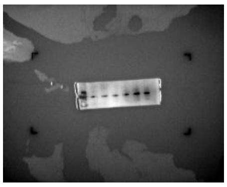	β-actin		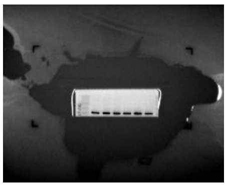
GDF15	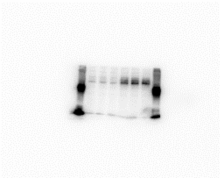	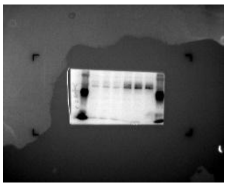	α-Tubulin		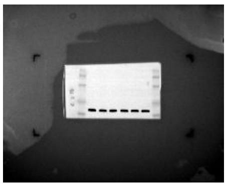
ERK1/2		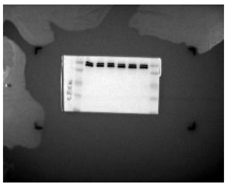	α-Tubulin		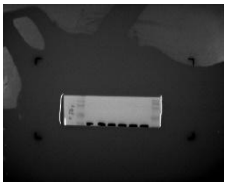
p-ERK1/2	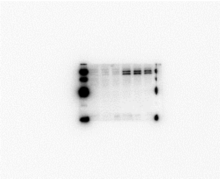	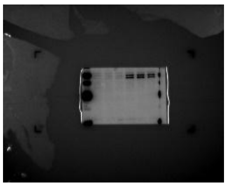	α-Tubulin		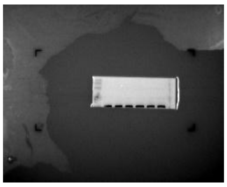
H2AX	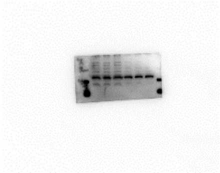	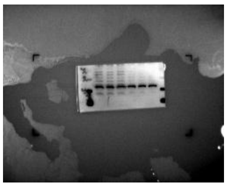	γH2AX	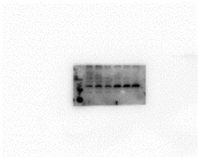	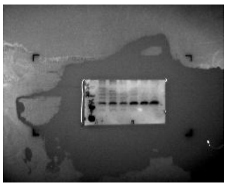
**siGDF15**					
GDF15	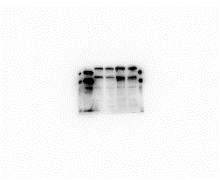	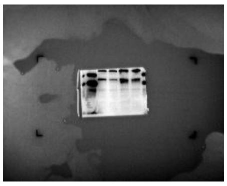	α-Tubulin	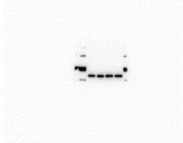	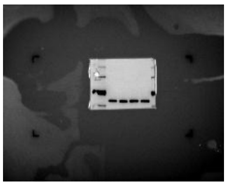

ERK1/2	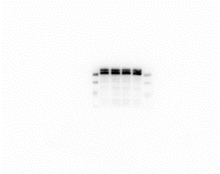	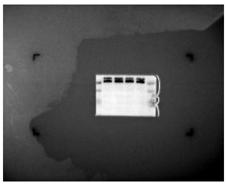	α-Tubulin	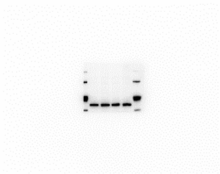	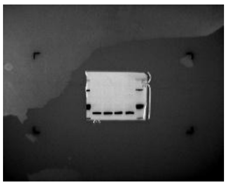
p-ERK1/2	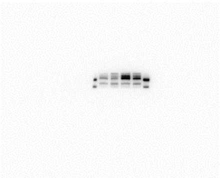	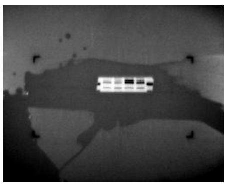	α-Tubulin		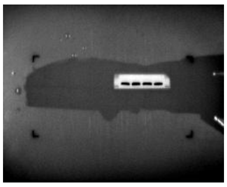
p16	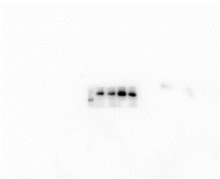	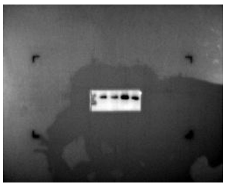	β-actin	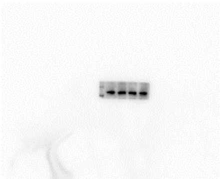	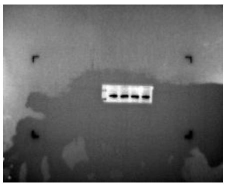
**U0126**					
ERK1/2		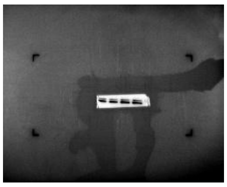	α-Tubulin	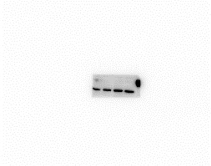	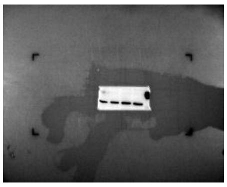
p-ERK1/2		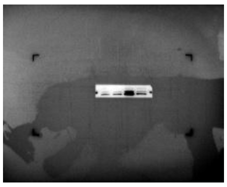	α-Tubulin	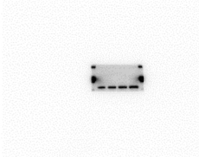	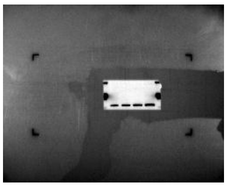
p16	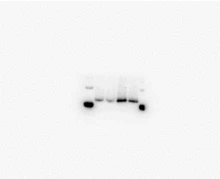	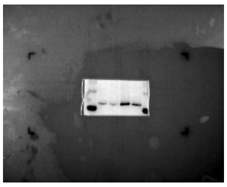	α-Tubulin	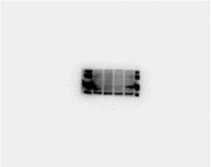	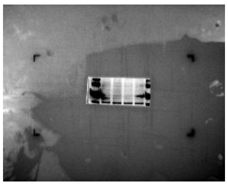
